# Supplementary material for: Immobilized Nucleoside 2′-Deoxyribosyltransferases from Extremophiles for Nucleoside Biocatalysis
Source: ACS Omega. 2024 Dec 30;10(1):1067–76. doi: 10.1021/acsomega.4c08364 (PMC11740241; doi:10.1021/acsomega.4c08364)
Supplement: Supplementary file 1 — ao4c08364_si_001.pdf [file ao4c08364_si_001.pdf]

Immobilized Nucleoside 2′-Deoxyribosyltransferases from Extremophiles for Nucleoside Biocatalysis

Saúl Antonio Hernández Martínez[a], Peijun Tang[b], Roberto Parra-Saldívar[c,d,], Elda M. Melchor-Martínez[c,], Clarissa Melo Czekster[b]*

a School of Engineering and Sciences, Tecnologico de Monterrey, 64849, Mexico.

b School of Biology, University of St Andrews, North Haugh, St Andrews, KY16 9ST, UK.

c Facultad de Medicina, Universidad Autónoma de Nuevo León, Monterrey, N.L., C.P. 64460, México.

d Megan Centre of Applied Mycology (MCAM), Faculty of Engineering and Applied Sciences, Cranfield University, Cranfield, Bedford, MK43 0AL, UK

*Email: cmc27@st-andrews.ac.uk

CONTENTS

Table S1. NDTs application for the synthesis of non-natural nucleosides, reaction conditions and HPLC retention times……………………………………………………………………….2

Figure S1. Structures for the enzyme activity assays: 1) 2′- Deoxyguanosine, 2) Guanosine, 3) 2′- Deoxyadenosine, 4) Adenosine, 5) 2′- Deoxythymidine, 6) Guanine, 7) Adenine, and 8) Thymine……….3

Figure S2. HPLC chromatogram for the biosynthesis of 2-fluoro-3′-deoxyadenosine catalyzed by immobilized CtNDT-DM……………………………………………………………………………...4

Figure S3. Mass Spectrometry for the biosynthesis of 2-fluoro-3′-deoxyadenosine catalyzed by immobilized CtNDT-DM...5

Figure S4. 19F NMR spectrum for 2-fluoroadenine standard 6

Figure S5. ^{19}F NMR spectrum for the biosynthesis of 2-fluoro-3′-deoxyadenosine catalyzed by immobilized *Ct*NDT-DM ……………………………………………………………………………7

Table S1. NDTs application for the synthesis of non-natural nucleosides, reaction conditions and HPLC retention times.

Enzyme	Reaction	Conditions	Compound	Retention time (min)
*Ct*NDT (1.8 μg)	2′- dGuo + Ade = Gua + 2′- dAdo	15 min, pH 8.5, 50° C, 180 rpm, 1 mL reaction.	2′- dGuo	3.8
			Ade	2.4
			Gua	1.4
			2′- dAdo	8.2
*Ct*NDT Y7F A9S (18 μg)	RGua + Ade = Gua + RAdo	15 min, pH 6.5, 55° C, 180 rpm, 1 mL reaction.	RGuo	3.2
			Ade	2.4
			Gua	1.4
			RAdo	8.0
*Bp*NDT (18 μg)	2′- dAdo + Thy = Ade + 2′- dThd	15 min, pH 8.0 25° C, 180 rpm, 1 mL reaction.	2′- dAdo	8.7
			Thy	2.2
			Ade	2.6
			2′- dThd	5.9

2′- dGuo (2′- Deoxyguanosine), Ade (Adenine), Gua (Guanine), 2′- dAdo (2′- Deoxyadenosine), RGua (Guanosine), RAdo (Adenosine), Thy (Thymine), and 2′- dThd (2′- Deoxythymidine). For structures FS1

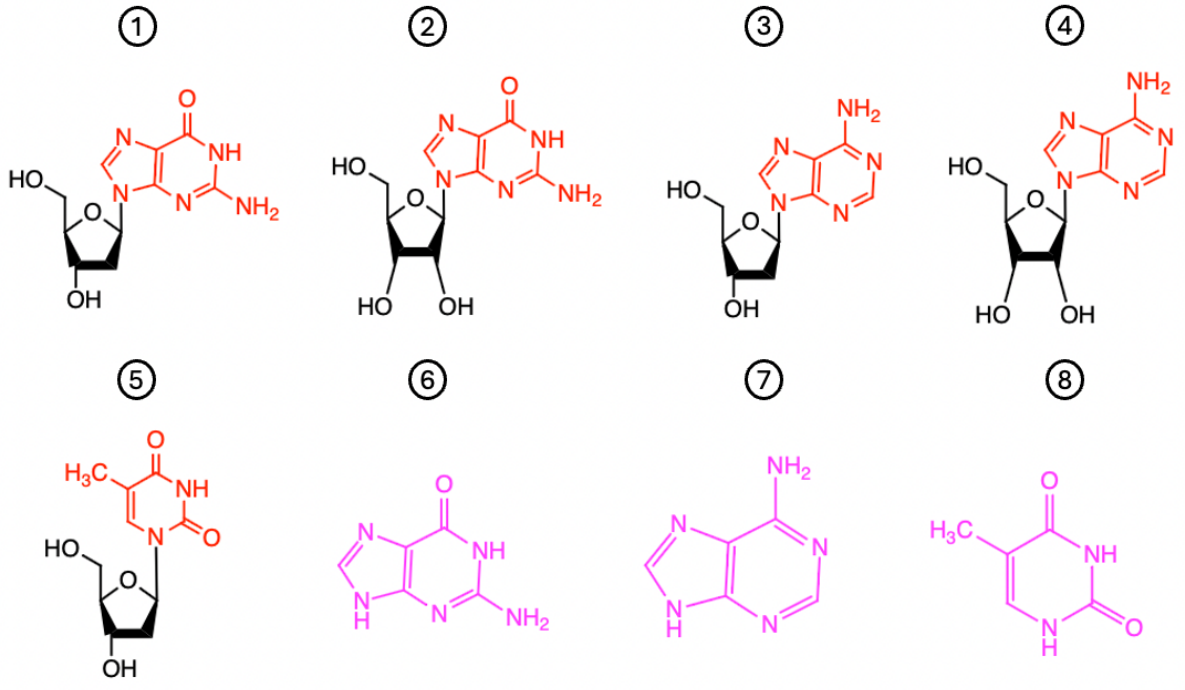

Figure S1. Structures for the enzyme activity assays: 1) 2′- Deoxyguanosine, 2) Guanosine, 3) 2′-Deoxyadenosine, 4) Adenosine, 5) 2′- Deoxythymidine, 6) Guanine, 7) Adenine, and 8) Thymine.

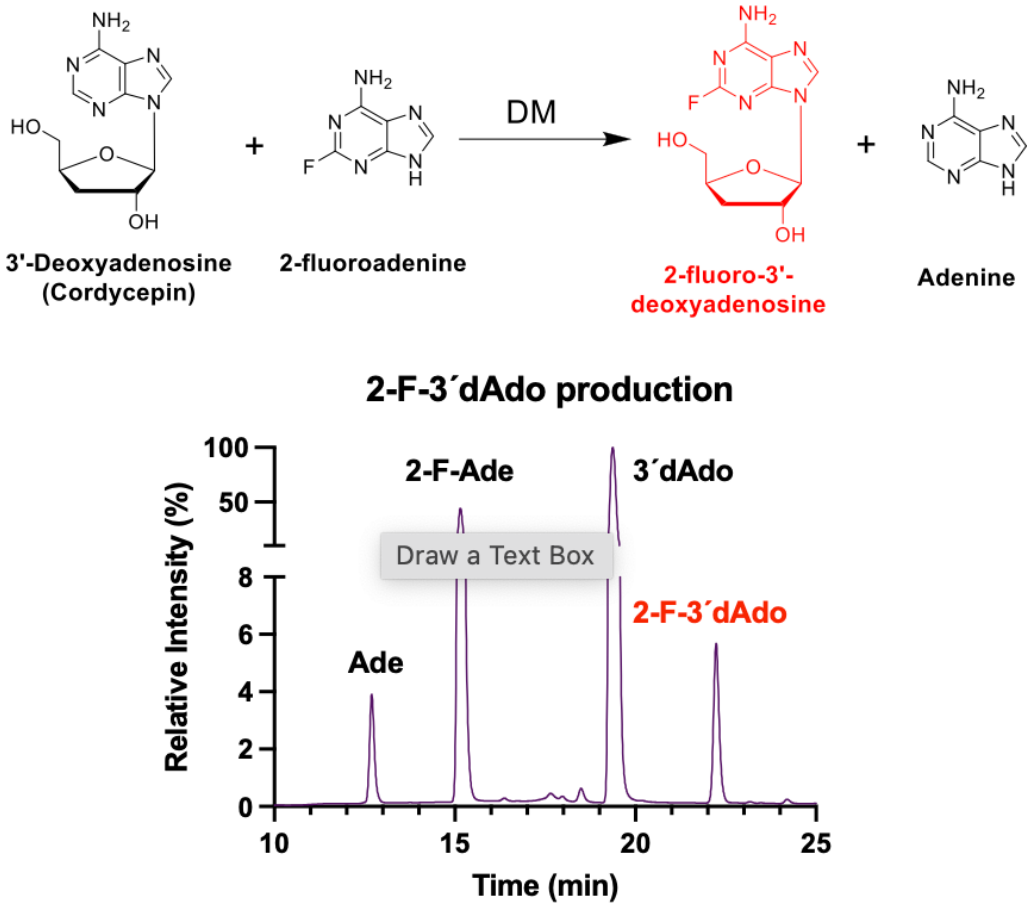

Figure S2. HPLC chromatogram for the biosynthesis of 2-fluoro-3′-deoxyadenosine catalyzed by immobilized CtNDT-DM. Retention times are as follows: 3′-deoxyadenosine (19.4 min), 2-fluoroadenine (15.2 min), adenine (12.7 min), and 2′-fluoro-3-deoxyadenosine (22.2 min).

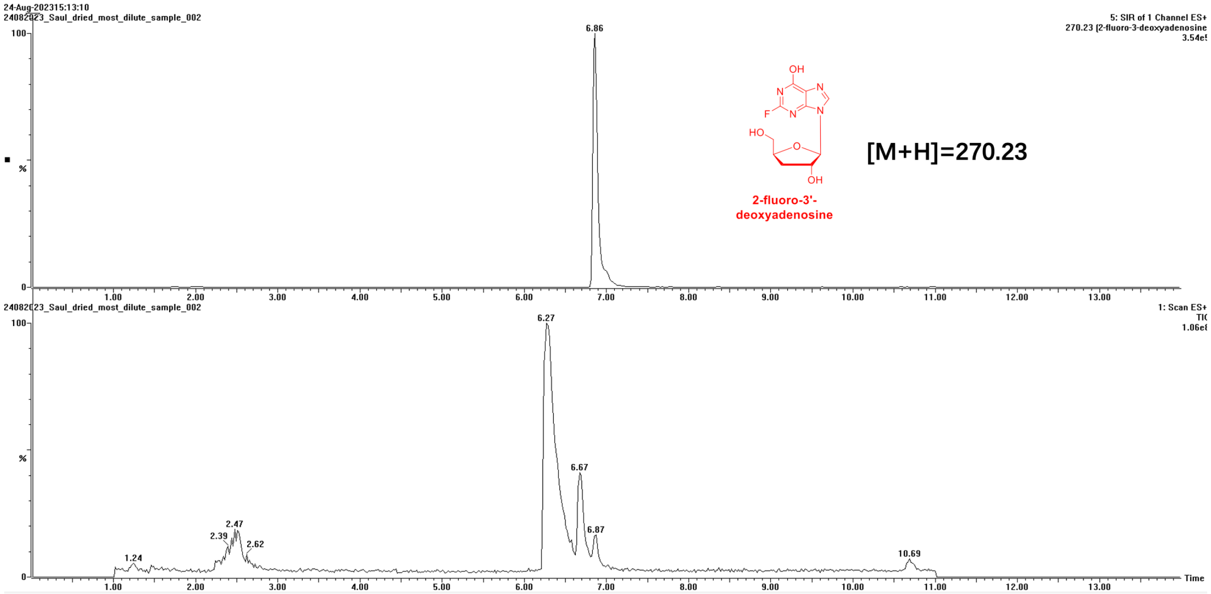

Figure S3. Mass Spectrometry for the biosynthesis of 2-fluoro-3′-deoxyadenosine catalyzed by immobilized CtNDT-DM. Top: single ion reaction monitoring (SIR) spectrum monitoring m/z = 270.23 (corresponding to 2-fluoro-3′-deoxyadenosine). Bottom, total ion counts (TIC) spectrum for the reaction.

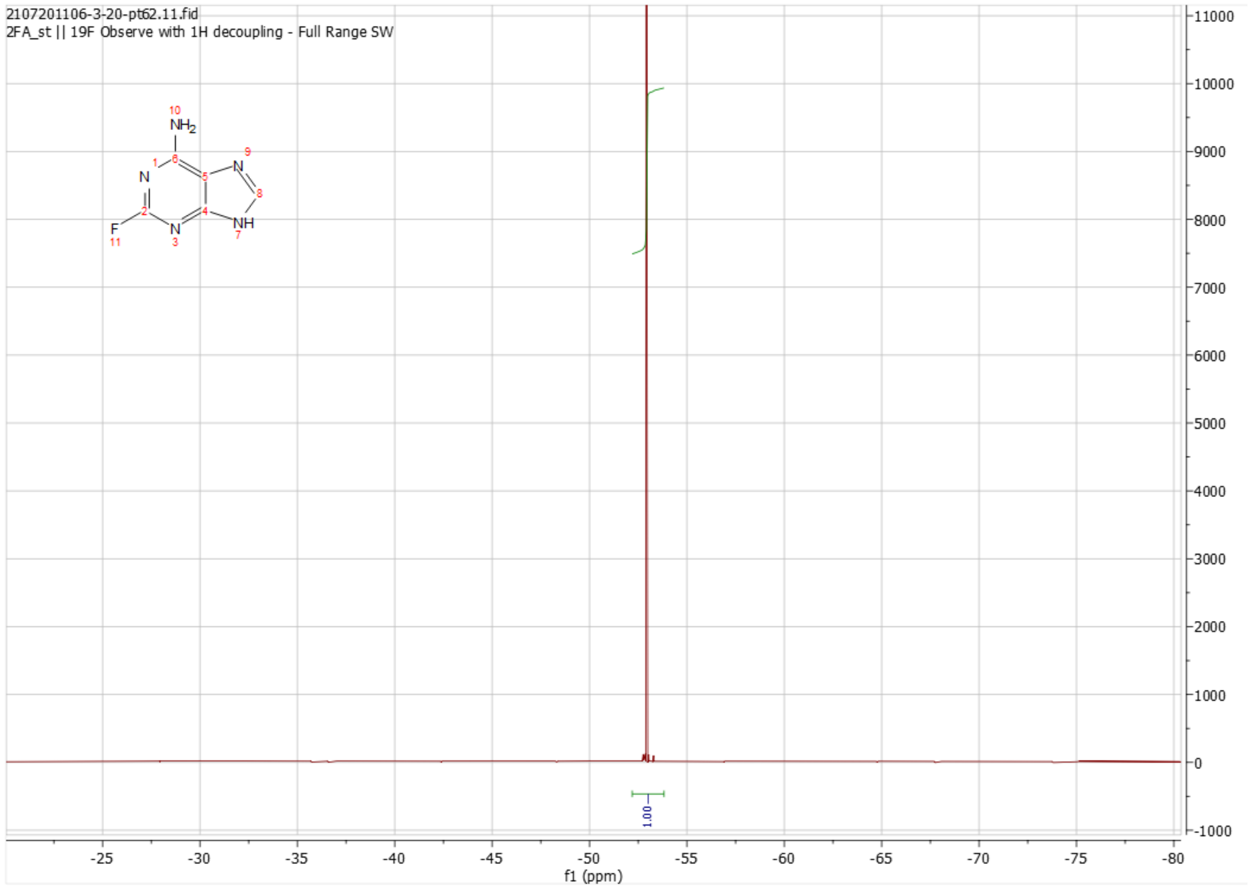

Figure S4. 19F NMR spectrum for 2-fluoroadenine standard.

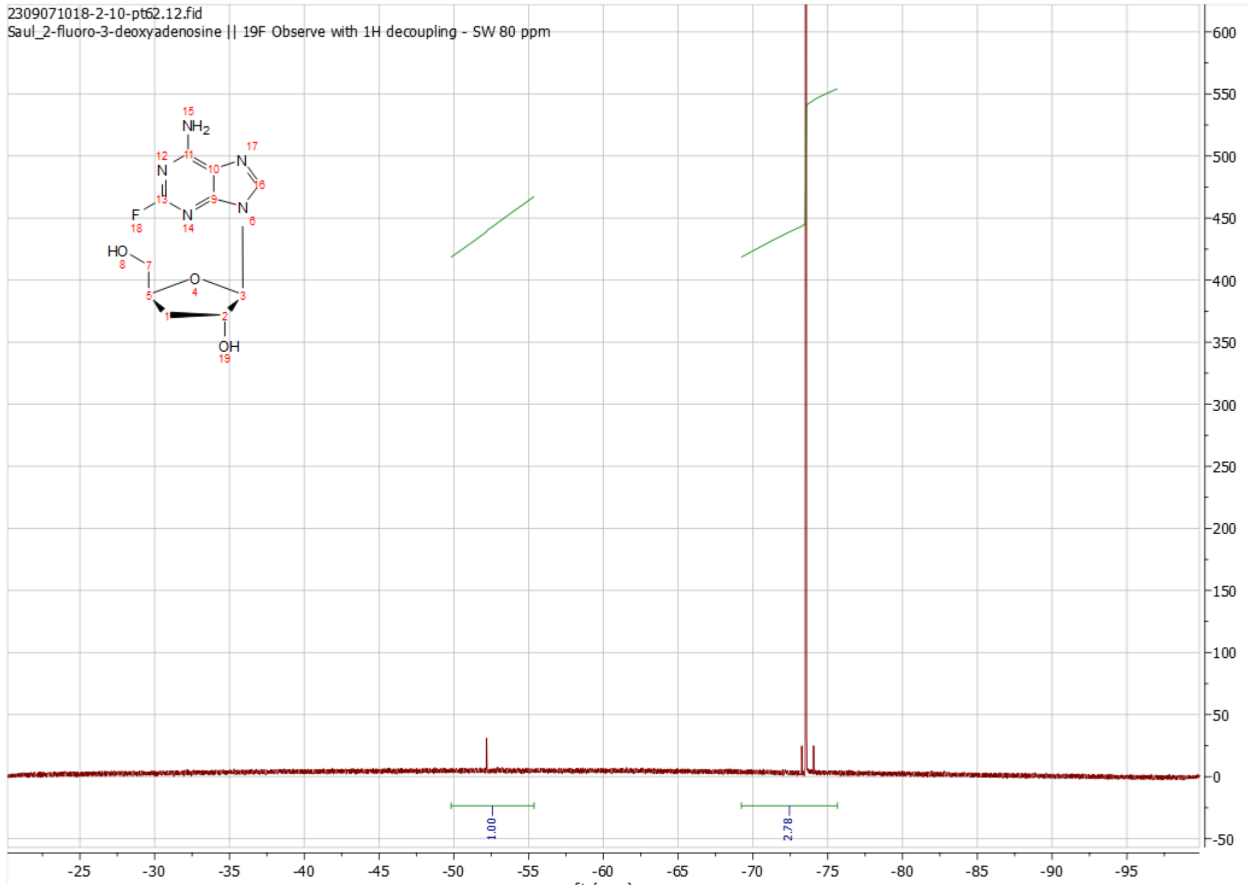

Figure S5. ^{19}F NMR spectrum for the biosynthesis of 2-fluoro-3′-deoxyadenosine catalyzed by immobilized *Ct*NDT-DM.
